# Development of hepatitis triggered by SARS-CoV-2 vaccination in patient with cancer during immunotherapy: a case report

**DOI:** 10.2217/imt-2021-0342

**Published:** 2022-06-13

**Authors:** Angioletta Lasagna, Marco Vincenzo Lenti, Irene Cassaniti, Paolo Sacchi

**Affiliations:** ^1^Medical Oncology Unit, Fondazione IRCCS Policlinico San Matteo, Pavia, 27100, Italy; ^2^Department of Internal Medicine, Clinica Medica, Fondazione IRCCS Policlinico San Matteo, University of Pavia, Pavia, 27100, Italy; ^3^Department of Microbiology & Virology, Molecular Virology Unit, Fondazione IRCCS Policlinico San Matteo, Pavia, 27100, Italy; ^4^Division of Infectious Diseases I, Fondazione IRCCS Policlinico San Matteo, Pavia, 27100, Italy

**Keywords:** autoimmune hepatitis, cancer patients, case report, COVID-19 vaccine, drug-induced liver injury, immune-related adverse events (irAEs), immunotherapy, HBV, mRNA vaccine, safety

## Abstract

Patients with cancer have a higher risk of severe COVID-19, and expert consensus advocates for COVID-19 vaccination in this population. Some cases of autoimmune hepatitis have been described after the administration of COVID-19 vaccine in the people in apparently good health. Immune checkpoint inhibitors (ICIs) are responsible for a wide spectrum of immune-related adverse events (irAEs). This article reports a case of hepatitis and colitis in a 52-year-old woman who was undergoing immunotherapy and was HBV positive 10 days after receiving the first Pfizer-BioNTech COVID-19 vaccine dose. Because both ICIs and the COVID-19 vaccines stimulate the immune response, the authors hypothesize that these vaccines may increase the incidence of irAEs during ICI treatment. There is a complex interplay between the immune-mediated reaction triggered by the vaccination and PD-L1 co-administration.

## Plain language summary

Patients with cancer have a higher risk of severe COVID-19, and expert consensus advocates for COVID-19 vaccination in this population. Some reports have described autoimmune hepatitis after the administration of COVID-19 vaccine. It is difficult, however, to establish a causal relationship between COVID-19 vaccination and autoimmune hepatitis. This article reports a case of hepatitis and colitis in a 52-year-old woman with lung cancer who was undergoing immunotherapy and was was found to be HBV positive 10 days after her first Pfizer-BioNTech COVID-19 vaccine dose. Because both immunotherapy and COVID-19 vaccines stimulate the immune response, the authors hypothesize that these vaccines may increase the incidence of immune-related side effects.

## Tweetable abstract

A complex interplay between the immune-mediated reaction triggered by mRNA #COVID19 vaccination and #PDL1 coadministration may rarely occur in patients with cancer during #immunotherapy

Patients with cancer have a well-known higher risk of severe COVID-19 [1]. After the emergence of the COVID-19 pandemic, caused by the severe acute respiratory syndrome coronavirus 2 (SARS-CoV-2), a large number of vaccines were in development. A recent paper reviewed cancer patients’ immune response to the main approved COVID-19 vaccines: the two mRNA vaccines (mRNA-1273 and the Pfizer-BioNTech vaccine) and the viral vector-based vaccines (ChAdOx1 nCov-19/AZD1222 and Ad26.COV2.S) [2]. In Italy, only the two mRNA vaccines have been approved for immunocompromised subjects, including the patients with cancer [3].

Expert consensus advocates for COVID-19 vaccination in the patients with cancer [4–6], although there are still many unclear issues about its safety and efficacy in terms of the magnitude and durability of the humoral and cell-mediated immune response in this frail population. A retrospective cohort study of 373 patients with cancer at a tertiary cancer center in London collected data of both solicited and unsolicited adverse events after at least one dose of COVID-19 vaccine. The authors showed that patients undergoing immune checkpoint inhibitor (ICI) treatment (15.3%) are less at risk of developing any vaccine-related adverse event (AE; odds ratio [OR]: 0.495; 95% CI: 0.256–00.958; p = 0.0037) [7]. The safety of COVID-19 vaccines in the patients with cancer, demonstrated in this study, is similar to the results of another observational study (SOAP-02) [8]: intriguingly, one patient during immunotherapy developed grade 4 transaminitis of undetermined cause 3 weeks after Pfizer/BioNTech COVID-19 vaccine.

In contrast, immune-related AEs (irAEs) caused by ICIs are better recognized. Usually, irAEs occur within few weeks after initiation of ICIs, but they have been documented 1  year after discontinuation of the therapy [9]. Skin AEs (rash, pruritus and vitiligo) are the most frequent irAEs (34–45%) [10], followed by colitis, hepatitis, pneumonitis and immune-related endocrinopathies [9]. A systematic review and meta-analysis of 15 studies showed that the incidence of all-grades diarrhea was 13.7% with PD-1 inhibitors and 35.4% with CTLA-4 inhibitors, and the incidence of all-grades colitis was 1.6 and 8.8%, respectively [11].

The incidence of all-grade immune-related acute hepatitis ranges between 4 and 9% of patients treated with anti-CTLA-4 and 18% of patients who received a combination of anti-PD-1 and anti-CTLA-4, whereas liver IRAEs are less frequent with anti-PD-1 alone, with an incidence of 1–4% [12].

## Case report

**Figure 1.**
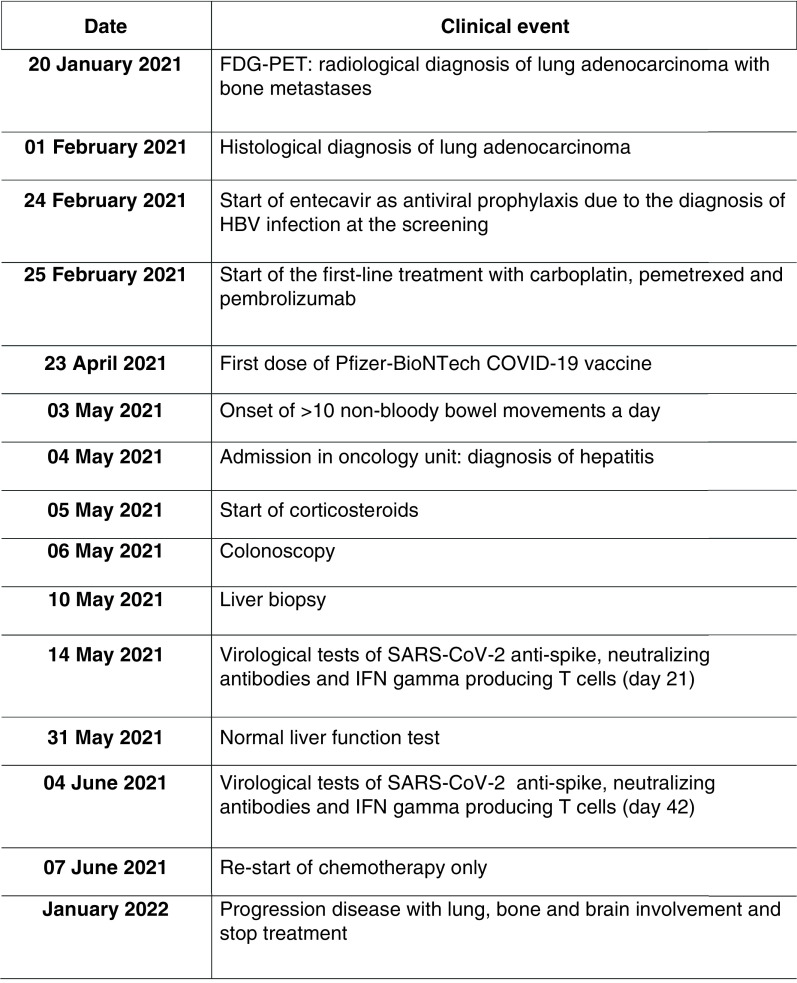
Timeline of clinical events.

The present report describes a case of hepatitis and colitis that occurred soon after a 52-year-old woman on immunotherapy received her first Pfizer-BioNTech COVID-19 vaccine dose (Figure 1). She had been diagnosed with lung adenocarcinoma with bone metastases; molecular analyses revealed that the tumor was negative for EGFR mutations and *ALK* gene rearrangements and that <1% of the tumor cells expressed PD‐L1. Accordingly, first‐line treatment with carboplatin, pemetrexed and pembrolizumab was started on 25 February 2021. Concurrent with the detection of cancer, hepatitis B virus (HBV) infection was diagnosed: HBsAg seropositive, HBV-DNA <20 IU/ml, HBeAg seronegative and anti-HBe positive, with normal liver function test. She received entecavir as antiviral prophylaxis before starting chemo-immunotherapy. Her medical history was unremarkable. No specific personal or family history suggested exposure to the major hepatotropic viruses.

After three cycles of therapy, the patient received the first dose of Pfizer-BioNTech COVID-19 vaccine without immediate side effects. After 10 days, she presented with >10 nonbloody bowel movements a day. The main laboratory data are summarized in Table 1. Abdominal ultrasound was unremarkable. Colon biopsies revealed microscopic colitis (lymphocytic subtype) with a concomitant eosinophilic infiltration, and liver histology showed only portal inflammation, without evidence of piecemeal necrosis and lobular hepatitis. Neither hepatocyte resetting nor plasma cell infiltrate was observed. Bile ducts were normal, and no cholestatic reaction was present.

**Table 1.** Laboratory tests at the time of the diagnosis of hepatitis and colitis.

Parameter	Value
WBC count (μl)	7100
Neutrophil (μl)	3900
NLR	2.29
CRP (mg/dl)	0.7
LDH (mg/dl)	356
AST (IU/l)	147
ALT (IU/l)	299
Total bilirubin (mg/dl)	1.98
GGT (IU/l)	139
Alkaline phosphatase (IU/l)	161
HAV - RNA	Negative
HBV – DNA (IU/ml)	<20 UI/ml (Abbot real-time PCR)
HCV – RNA	Negative
HDV – RNA	Negative
HEV – RNA	Negative
CMV IgG (U/ml)	<12 (>14 positive)
CMV IgM (U/ml)	<18 (>22 positive)
EBV IgG (U/ml)	<20 (<20 negative)
EBV IgM (U/ml)	<20 (<20 negative)
ANA	<1:80
S-Ama	<1:40
PR3-ANCA	Negative
MPO-ANCA	Negative
Serum IgG (mg/dl)	1400
Fecal calprotectin (ng/mg)	591
*Clostridium difficile* toxins (A and B)	Negative
Stool cultures for bacteria, ova and parasites	Negative

ALT: Alanine aminotransferase; ANA: Antinuclear antibody; AST: Aspartate aminotransferase; CMV: Cytomegalovirus; CRP: C-reactive protein; EBV: Epstein-Barr virus; GGT: Gamma-glutamyl transferase; HAV: Hepatitis A virus; HBV: Hepatitis B virus; HCV: Hepatitis C virus; HDV: Hepatitis Delta virus; HEV: Hepatitis E virus; LDH: Lactate dehydrogenase; MPO-ANCA: Myeloperoxidase anti-neutrophil cytoplasmic antibody; NLR: Neutrophil/lymphocyte ratio; S-Ama: Anti-smooth-muscle antibody; PR3-ANCA: Protenase-3anti-neutrophil cytoplasmic antibody; WBC: White blood cell.

The Revised Original Score for autoimmune hepatitis pretreatment [13] was 2.

The corticosteroid treatment with high-dose prednisone (1 mg/kg) was started with the rapid normalization of liver enzymes and the improvement of diarrhea. This case was notified to the Italian Sanitary Authority (Agenzia Italiana del Farmaco [AIFA]), and the patient resumed only chemotherapy. The main laboratory data at the resolution of irAEs are summarized in Table 2.

**Table 2.** Laboratory tests at the resolution of hepatitis and colitis.

Parameter	Value
WBC count (μl)	7000
Neutrophil (μl)	5100
NLR	3
CRP (mg/dl)	0.3
LDH (mg/dl)	267
AST (IU/l)	27
ALT (IU/l)	31
Total bilirubin (mg/dl)	0.95
GGT (IU/l)	40
Alkaline phosphatase (IU/l)	118
HAV – RNA	Negative
HBV – DNA (IU/ml)	<20 (Abbot real-time PCR)
HCV – RNA	Negative
HDV – RNA	Negative
HEV – RNA	Negative

ALT: Alanine aminotransferase; AST: Aspartate aminotransferase; CRP: C-reactive protein; GGT: Gamma-glutamyl transferase; HAV: Hepatitis A virus; HBV: Hepatitis B virus; HCV: Hepatitis C virus; HDV: Hepatitis Delta virus; HEV: Hepatitis E virus; LDH: Lactate dehydrogenase; NLR: Neutrophil/lymphocyte ratio; WBC: White blood cell.

Notably, 3 weeks after the first dose, the patient developed a positive response in terms of both humoral and cell-mediated response. Despite the absence of a second dose of vaccine, the immune response remained sustained after 21 days of follow-up (Table 3).

**Table 3.** Values of SARS-CoV-2 anti-spike, neutralizing antibodies and IFN-γ producing T cells at days 21 and 42 after the first dose of Pfizer-BioNTech COVID-19 vaccine.

Parameters day 21	Value (range)
S1/S2 IgG level (AU/ml)	89.5 (cutoff >15 AU/ml)
SARS-CoV-2 neutralizing antibodies	1:40 (cutoff 1:10)
Spike-specific ELIspot assay	20 IFN-γ-producing T cells (cutoff 10)
**Parameters day 42**	**Value (range)**
S1/S2 IgG level (AU/ml)	128 (cutoff >15 AU/ml)
SARS-CoV-2 neutralizing antibodies	1:40 (cutoff 1:10)
Spike-specific ELIspot assay	85 IFN-γ-producing T cells (cutoff 10)

**Figure 2.**
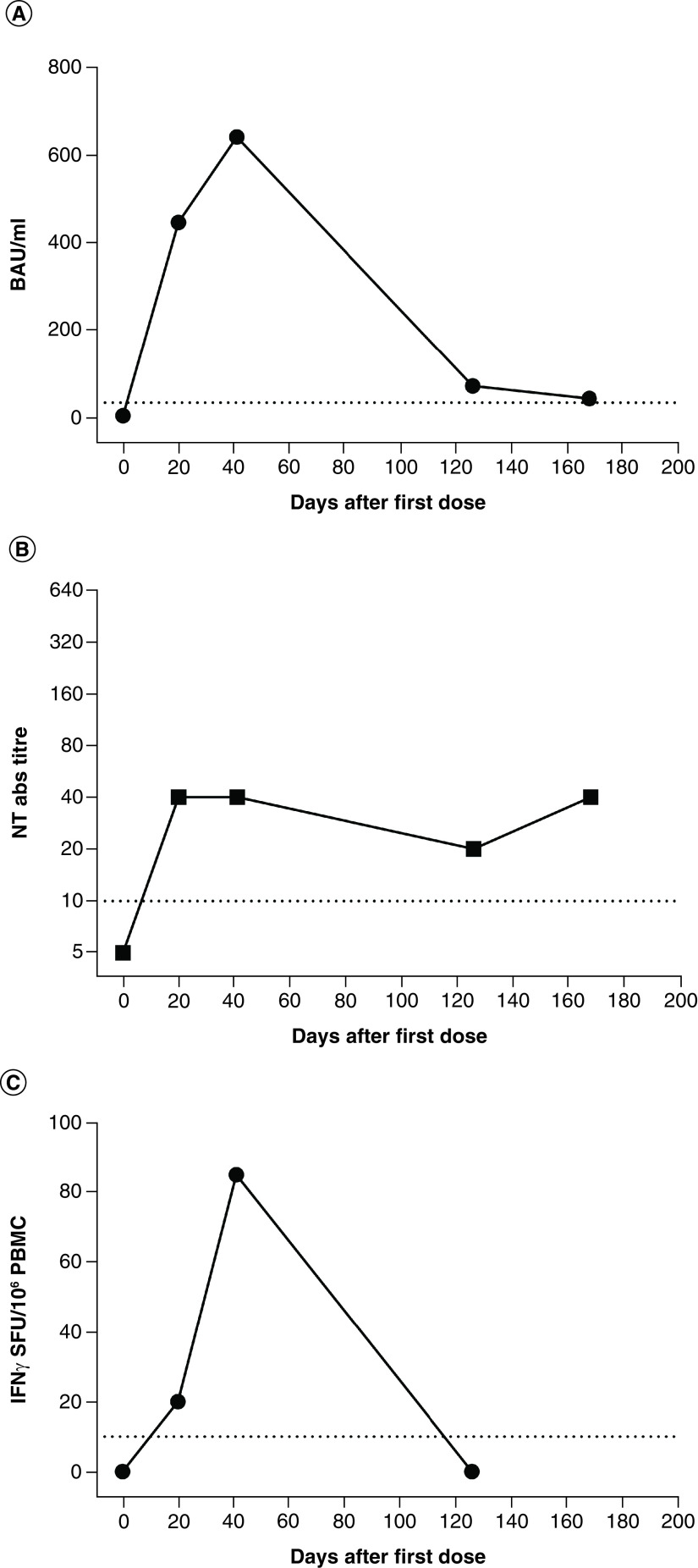
Humoral and cell-mediated response after Pfizer-BioNTech COVID-19 vaccine. Anti-spike IgG **(A)**, NT Abs **(B)** and spike-specific T-cell response **(C)** were measured in the patient at the baseline, 21 days after the first dose of COVID-19 vaccine and after 3 and 6 months. Ab: Antibody; BAU: Binding antibody unit; NT: Neutralizing; PBMC: Peripheral blood mononuclear cell.

For this reason, it was decided to omit the second dose of COVID-19 vaccine and to recheck her humoral and cell-mediated response after 3 and 6 months with the evidence of the persistence of only humoral response (Figure 2).

## Discussion

As previously reported, some cases of liver toxicity with the characteristics of autoimmune hepatitis have been described after the administration of COVID-19 vaccine. Lodato and colleagues [14] reported severe cholestatic hepatitis developed in a 43-year-old woman a few days after the second dose of Pfizer-BioNTech COVID-19 vaccine. The liver biopsy demonstrated the presence of eosinophil infiltrate with the absence of autoantibodies, but the patient had a dramatic response to steroid treatment, similar to autoimmune hepatitis. Other analogue cases were described by Bril [15] and Londoño [16] – in a 35-year-old woman in her third month postpartum 1 week after the first dose of Pfizer-BioNTech COVID-19 vaccine and in a 41-year-old woman after her first dose of mRNA-1273 vaccine, respectively. They were apparently in good health condition. Antinuclear antibody was positive in both cases, whereas the anti-smooth-muscle antibody was positive and serum IgG level was raised only in the latter case. Clayton-Chubb *et al.* [17] described a case of vaccine-induced autoimmune hepatitis (AIH) after COVID-19 vaccination in a 36-year-old Iraqi-born man without apparent confounding factors. In contrast to the previous cases, this patient had received the Oxford-AstraZeneca vaccine, an adenovirus-based vaccine. Chow *et al.* [18] recently conducted a systematic search of the literature about the documented cases of AIH following COVID-19 vaccination [14–17,19–30]. They reported that 29 patients received mRNA vaccines and three patients received the Oxford-AstraZeneca vaccine. None of these patients had solid tumors, and only 10 had a history of liver disease [18]. More recently, Pinazo-Bandera *et al* described two new cases of AIH related to COVID-19 vaccination [31] and suggested that regulatory authorities should include this potential AE on the label of COVID-19 vaccines. A summary of the reported cases of vaccine-induced AIH is shown in Table 4

**Table 4.** Autoimmune hepatitis and COVID-19 vaccination.

First author, year	Sex	Age	Type of COVID-19 vaccine	Onset timing	Personal history	Autoimmune study	Hepatotropic viruses	Liver biopsy	Steroids (yes/no)	Outcome	Ref.
Lodato, 2021	F	43	Pfizer-BioNTech	After the second dose	Venous insufficiency and mild dyslipidemia with intermittent ALT increase	ANA negative S-Ama negative MPO-ANCA negative	HAV negative HBV negative HCV negative CMV negative EBV negative	Moderate portal inflammatory infiltrate and interface hepatitis in the portal tract	Yes	Resolution at 8 weeks	[14]
Bril, 2021	F	35	Pfizer-BioNTech	After the first dose	Third month postpartum	ANA positive S-Ama negative	HAV negative HBV negative HCV negative CMV negative EBV negative	Intense lymphoplasmacytic infiltrate effacing the interface with rosette formation	Yes	Resolution at 2 months	[15]
Londoño, 2021	F	41	mRNA-1273 (Moderna)	After the second dose	Premature ovarian failure and substitutive hormonal therapy	ANA positive S-Ama positive anti-SLA positive	HAV negative HBV negative HCV negative CMV negative EBV negative	Severe interface hepatitis and lobular inflammation	Yes	Resolution	[16]
Clayton-Chubb, 2021	M	36	ChAdOx1 nCoV-19 vaccine (Oxford-AstraZeneca)	After the first dose	Hypertension	ANA positive	HAV negative HBV negative HCV negative CMV negative EBV negative	Interface hepatitis with a mixed inflammatory cell infiltrate	Yes	Resolution	[17]
Garrido, 2021	F	65	mRNA-1273 (Moderna)	After the first dose	JAK2 V617F-positive polycythemia vera	ANA positive	HAV negative HBV negative HCV negative CMV negative EBV negative	Intense lymphoplasmacytic infiltrate and interface hepatitis	Yes	Resolution	[19]
Ghielmetti, 2021	M	63	mRNA-1273 (Moderna)	After the first dose	Type 2 diabetes and ischemic heart disease	ANA positive	HAV negative HBV negative HCV negative	Inflammatory portal infiltrate with interface hepatitis	Yes	Resolution	[20]
Goulas, 2022	F	52	mRNA-1273 (Moderna)	After the first dose	None	ANA positive S-Ama positive	HAV negative HBV negative HCV negative CMV negative EBV negative	Severe inflammatory infiltration	Yes	Resolution	[21]
McShane, 2021	F	71	mRNA-1273 (Moderna)	After the first dose	Osteoarthritis of the knees	S-Ama positive	HBV negative HCV negative CMV negative EBV negative HAV IgG positive HAV IgM negative	Interface hepatitis	Yes	Resolution	[22]
Palla, 2022	F	40	Pfizer-BioNTech	After the second dose	Sarcoidosis	ANA positive S-Ama negative	HBV negative HCV negative CMV negative EBV negative	Interface necro-inflammation and severe lobular inflammatory infiltration	Yes	Resolution	[23]
Rela, 2021	1° F 2° M	1° 38 2° 62	1° ChAdOx1 nCoV-19 vaccine (Oxford-AstraZeneca) 2° ChAdOx1 nCoV-19 vaccine (Oxford-AstraZeneca)	1° After the first dose 2° After the first dose	1° Hypothyroidism 2° None	1° ANA positive S-Ama negative anti-SLA negative 2° ANA negative S-Ama negative anti-SLA negative	1° HAV negative HBV negative HCV negative 2° HAV negative HBV DNA negative HBcAb positive HCV negative	1° multiacinar hepatic necrosis and periportal neocholangiolar proliferation 2° Portal/periportal neocholangiolar proliferation and mild to moderate inflammation	1° Yes 2° Yes	1° Resolution 2° Death	[24]
Rocco, 2021	F	80	Pfizer-BioNTech	After the second dose	Hashimoto's thyroiditis	ANA positive S-Ama negative anti-SLA negative	HAV negative HBV negative HCV negative CMV negative EBV negative	Interface hepatitis with a moderate degree of lymphoplasmacytic infiltrate	Yes	Resolution	[25]
Shroff, 2021	1° M 2° F 3° M 4° M 5° F 6° M 7° F 8° F 9° F 10° F 11° M 12° F 13° F 14° F 15° F 16° M	1° 46 2° 61 3° 61 4° 71 5° 74 6° 73 7° 25 8° 61 9° 37 10° 33 11° 68 12° 70 13° 66 14° 68 15° 59 16° 65	1° Pfizer-BioNTech 2° Pfizer-BioNTech 3° Pfizer-BioNTech 4° Pfizer-BioNTech 5° Pfizer-BioNTech 6° Pfizer-BioNTech 7° Pfizer-BioNTech 8° Pfizer-BioNTech 9° Pfizer-BioNTech 10° Pfizer-BioNTech 11° Pfizer-BioNTech 12° Pfizer-BioNTech 13° mRNA-1273 (Moderna) 14° mRNA-1273 (Moderna) 15° mRNA-1273 (Moderna) 16° mRNA-1273 (Moderna)	1° After the first dose 2° After the second dose 3° After the second dose 4° After the second dose 5° After the second dose 6° After the first dose 7° After the first dose 8° After the second dose 9° After the first dose 10° After the second dose 11° After the first dose 12° After the second dose 13° After the first dose 14° After the first dose 15° After the second dose 16° After the second dose	1° NAFLD 2° None 3° None 4° Compensated cirrhosis, HCV treated 5° Extramedullary hematopoiesis of unknown significance 6° AIH 7° None 8° None 9° None 10° AIH treated and Compensated cirrhosis 11° AIH treated and Compensated cirrhosis 12° Prior biliary stricture after cholecystectomy 13° AIH treated 14° None 15° None 16° None	1° S-Ama 1:40 2° S-Ama 1:160 3° ANA negative S-Ama negative anti-SLA negative 4° None performed 5° ANA positive 6° None performed 7° ANA 1:640 8° ANA 1:320 9° ANA negative S-Ama negative anti-SLA negative 10° None performed 11° None performed 12° None performed 13° None performed 14° ANA negative S-Ama negative anti-SLA negative 15° ANA 1:640 14° ANA negative S-Ama negative anti-SLA negative	1° Viral serology negative 2° Viral serology negative 3° Viral serology negative 4° None performed 5° Viral serology negative 6° None performed 7° Viral serology negative 8° EBV viral load 78 9° Viral serology negative 10° None performed 11° None performed 12° None performed 13° Viral serology negative 14° Viral serology negative 15° EBV VCA IgM+, IgG+ Viral serology negative 16° Viral serology negative	1° Portal inflammation; No interface hepatitis 2° Portal inflammation; No interface hepatitis 3° Portal inflammation; No interface hepatitis 4° None performed 5° None performed 6° None performed 7° None performed 8° Portal inflammation; No interface hepatitis 9° None performed 10° Portal inflammation and interface hepatitis 11° Portal inflammation and interface hepatitis 12° None performed 13° Portal inflammation with interface hepatitis 14° Portal inflammation; No interface hepatitis 15° Portal inflammation; No interface hepatitis 16° Portal inflammation; No interface hepatitis	1° No 2° Yes 3° No 4° No 5° No 6° Yes 7° No 8° Yes 9° No 10° Yes 11° Yes 12° No 13° Yes 14° Yes 15° Yes 16° No	1° Resolution 2° Resolution 3° Resolution 4° Resolution 5° Resolution 6° Resolution 7° Resolution 8° Resolution 9° Resolution 10° Resolution 11° Resolution 12° Resolution 13° Resolution 14° Resolution 15° Resolution 16° Resolution	[26]
Tan, 2021	F	56	mRNA-1273 (Moderna)	After the first dose	Dyslipidemia	ANA positive S-Ama positive	HAV negative HBV negative HCV negative CMV negative EBV negative	Portal inflammation with interface hepatitis	Yes	Resolution	[27]
Tun, 2021	M	47	mRNA-1273 (Moderna)	After the second dose	None	None performed	HAV negative HBV negative HCV negative CMV negative EBV negative	Portal inflammation	Yes	Resolution	[28]
Vuille-Lessard, 2021	F	76	mRNA-1273 (Moderna)	After the first dose	Hashimoto thyroiditis and prior urothelial carcinoma	ANA 1:1280 S-Ama 1:1280	HAV negative HBV negative HCV negative CMV negative	Portal inflammation with interface hepatitis	Yes	Resolution	[29]
Zhou, 2022	F	36	mRNA-1273 (Moderna)	After the first dose	Ulcerative colitis and PSC	ANA 1:2,560	HAV negative HBV negative HCV negative CMV negative EBV negative	Portal inflammation with interface hepatitis	Yes	Resolution	[30]
Pinazo-Bandera, 2022	1° F 2° M	1° 77 2° 23	1° Pfizer-BioNTech 2° mRNA-1273 (Moderna)	1° After the first dose 2° After the second dose	1° Hypertension 2° None	1° ANA 1: 160 2° ANA negative S-Ama negative	1° - 2° Viral serology negative	1° Portal inflammation 2° Portal inflammation	1° Yes 2° Yes	1° Resolution 2° Resolution	[31]

°Indicates patient number.

AIH: Autoimmune hepatitis; ANA: Antinuclear antibody; CMV: Cytomegalovirus; EBV: Epstein-Barr virus; F: Female; HAV: Hepatitis A virus; HBV: Hepatitis B virus; HBcAb: HBV core antibody; HCV: Hepatitis C virus; HDV: Hepatitis Delta virus; HEV: Hepatitis E virus; M: Male; MPO-ANCA: Myeloperoxidase anti-neutrophil cytoplasmic antibody; anti-SLA: anti-soluble liver antigen; NAFLD: Nonalcoholic fatty liver disease; PR3-ANCA: Protenase-3anti-neutrophil cytoplasmic antibody; PSC: Primary sclerosing cholangitis; S-Ama: Anti-smooth-muscle antibody; VCA: Viral capsid antigen.

These cases indicate the need to recognize and promptly treat this unusual side effect irrespective of the mechanism of action of the vaccines.

The present case is unusual for the co-occurrence of two immune-mediated reactions: mixed eosinophilic/lymphocytic colitis and alanine aminotransferase (ALT) elevation. We have performed a complex diagnostic pathway to evaluate HBV reactivation, autoimmune hepatitis and a drug-induced liver injury related to ICI administration.

First, the authors hypothesized that the HBV reactivation was induced by anti-PD-1/PD-L1. The blocking of the PD-1/PD-L1 axis may lead to the destruction of hepatocytes with the release of previously latent virus into the circulation and promote proliferation of Tregs with consequent increased immunosuppression, and hence the reactivation of HBV [32]. The patient was chronically infected with HBV, but the HBV-DNA remained unquantifiable, so this hypothesis was discarded.

Second, immune-mediated toxicity was considered. Colitis is the second most commonly reported AE with ICI administration, and the symptoms typically develop from 6 to 8 weeks from the start of treatment; median onset of transaminase elevation is approximately 6–14 weeks after starting ICIs [33]. The pathogenesis of ICI-induced hepatitis is not well understood. Two recent papers point out the immunological mechanism in animal models [34,35]: macrophages and neutrophils are mediators and effectors of aberrant inflammation in TH1-promoting immunotherapy, suggesting distinct mechanisms of toxicity and antitumor immunity.

## What role might the vaccine have played?

The mRNA vaccine strongly stimulates innate immunity by the immunostimulatory properties of mRNA, which triggers intracellular innate sensors, including Toll-like receptors 3 and 7 and components of the inflammasome, resulting in the production of interferon I and other pro-inflammatory cytokines and chemokines [36]. This mechanism is the basis of the immunologic activation leading to the neutrophil-driven liver damage, and it has been recognized as a probable effector of immune-mediated hepatitis following ICI administration. Ultimately, the clinical evolution was consistent with an immune-mediated side effect of ICIs, but the vaccination may have triggered such toxicity.

In a cohort study, none of the 134 patients enrolled who had received two doses of the BNT162b2 COVID-19 vaccine reported any severe irAE [37]. Because both ICIs and COVID-19 vaccines stimulate the immune response, it has been hypothesized that these vaccines may increase the incidence of the immune-related AEs with ICI treatment. To date, there are no data demonstrating a direct answer.

## Conclusion

This case report may represent the first to describe a complex interplay between an immune-mediated reaction triggered by vaccination and PD-L1 co-administration. It may be interesting for clinicians involved in the management of cancer patients who receive COVID-19 vaccination. The main limit of this report is that the diagnosis was one of exclusion; it is therefore not possible to define the exact role played by the various drugs (ICIs and COVID-19 vaccine). Importantly, this report should not deter individuals from getting vaccinated but only increase awareness of possible pharmacological interactions.

**Summary points**

- Expert consensus advocates for cancer patients to be vaccinated against SARS-CoV-2.
- In a retrospective cohort study of 373 cancer patients, the authors showed that patients undergoing immunotherapy (immune checkpoint inhibitors [ICIs]) are at less risk of developing any vaccine-related adverse events.
- This report describes a case of hepatitis and colitis in a 52-year-old woman with lung adenocarcinoma who was receiving ICIs; 10 days after her first Pfizer-BioNTech COVID-19 vaccine dose, she was HBV positive.
- Some cases of liver toxicity with characteristics of autoimmune hepatitis have been described after administration of COVID-19 vaccine in people who are in apparent good health.
- The mRNA vaccine strongly stimulates innate immunity through the immunostimulatory properties of mRNA, which triggers intracellular innate sensors. This mechanism is the basis of the immunologic activation leading to neutrophil-driven liver damage, and it has been recognized as a probable effector of immune-mediated hepatitis after ICI administration.
- This case report describes a complex diagnostic pathway to evaluate HBV reactivation, autoimmune hepatitis and a drug-induced liver injury related to ICI administration.
